# Genomic Analysis and Characterization of *Pseudotabrizicola formosa* sp. nov., a Novel Aerobic Anoxygenic Phototrophic Bacterium, Isolated from Sayram Lake Water

**DOI:** 10.3390/microorganisms10112154

**Published:** 2022-10-30

**Authors:** Yu-Qi Ye, Ji-Ru Han, Jin-Xin Zhao, Meng-Qi Ye, Zong-Jun Du

**Affiliations:** 1 Marine College, Shandong University, Weihai 264209, China; 2 The Walter and Eliza Hall Institute of Medical Research, Parkville, VIC 3052, Australia; 3 Infection Program and Department of Microbiology, Monash Biomedicine Discovery Institute, Monash University, Clayton, VIC 3800, Australia; 4 Weihai Research Institute of Industrial Technology of Shandong University, Weihai 264209, China

**Keywords:** *Pseudotabrizicola formosa* sp. nov., *Pseudotabrizicola*, polyphasic taxonomy, lake water, aerobic anoxygenic phototrophic bacteria, anoxygenic photosystem II

## Abstract

Aerobic anoxygenic photosynthetic bacteria (AAPB) are a kind of heterotrophic prokaryote that can use bacteriochlorophyll (BChl) for photosynthesis without oxygen production and they are widely distributed in aquatic environments, including oceans, lakes, and rivers. A novel aerobic anoxygenic photosynthetic bacterium strain XJSP^T^ was isolated during a study of water microbial diversity in Sayram Lake, Xinjiang Province, China. Strain XJSP^T^ was found to grow optimally at 33 °C, pH 7.5 with 1.0% (*w*/*v*) NaCl, and to produce bacteriochlorophyll a and carotenoids. Phylogenetic analysis based on 16S rRNA gene sequence and concatenated alignment sequences of 120 ubiquitous single-copy proteins both supported that strain XJSP^T^ belonged to the genus *Pseudotabrizicola*. Both average nucleotide identity (ANI) and DNA–DNA hybridization (DDH) values were below the species delineation threshold. The primary polar lipids were phosphatidylcholine, phosphatidylglycerol, phosphatidylethanolamine, one unknown lipid, and one unidentified phospholipid. Based on the results of polyphasic analyses performed in this study, strain XJSP^T^ represents a new member of the genus *Pseudotabrizicola*, for which the name *Pseudotabrizicola formosa* sp. nov. is proposed. The type strain is XJSP^T^ (= KCTC 52636^T^ = MCCC 1H00184^T^ = SDUM 107003^T^). Comparative genomic analysis showed that four species of the genus *Pseudotabrizicola* shared 2570 core genes and possessed a complete anoxygenic photosystem II.

## 1. Introduction

Aerobic anaerobic photosynthetic bacteria (AAPB) are widely distributed in aquatic environments (oceans, lakes, and rivers). As a heterotrophic group, they can use reduced organic matter as electron donor under aerobic conditions to carry out non-oxygen-producing photosynthesis, relying on unique bacteriochlorophyll (BChl) and light reaction center [1,2]. The ATP produced by photosynthesis replenishes the energy required for growth, not only reducing the consumption of organic carbon, but also increasing the amount of dissolved organic carbon (DOC) entering the cells, which is vital for biogeochemical cycles [3,4,5]. 

The genus *Tabrizicola*, belonging to the family *Rhodobacteraceae* in the class *Alphaproteobacteria*, was firstly proposed by Vahideh et al. in 2013 and consists of eight validly published species and three effectively described species at the time of writing [6]. In 2022, Ma et al. reclassified *Tabrizicola sediminis*, *Tabrizicola alkalilacus*, and *Tabrizicola algicola* into a novel genus, *Pseudotabrizicola* gen. nov., as *Pseudotabrizicola sediminis* comb. nov., *Pseudotabrizicola alkalilacus* comb. nov., and *Pseudotabrizicola algicola* comb. nov. according to the results of polyphasic investigations [7]. Existing members of the genus *Pseudotabrizicola* are Gram-strain-negative, catalase- and oxidase-positive, and have Q-10 as the main respiratory quinone. Moreover, *P. sediminis* KCTC 72015^T^ and *P. algicola* KCTC 72206^T^ were reported to belong to AAPB, a kind of heterotrophic bacteria which have a photosynthetic gene and can produce BChl a in aerobic condition but cannot grow photoautotrophically under anaerobic conditions [8,9,10]. 

During our research of bacterial diversity at Sayram Lake, a cream-colored bacterium designated XJSP^T^ was isolated from a lake water sample using a dilution-plating procedure and conventional isolation techniques. Polyphasic taxonomic investigations, including phenotypic characterizations, chemotaxonomic properties, and phylogenetic analysis, showed that strain XJSP^T^ was a novel aerobic anoxygenic phototrophic bacterium species affiliated to the genus *Pseudotabrizicola*. 

## 2. Materials and Methods 

### 2.1. Bacterial Isolation and Culture

Samples from various habitats were gathered for bacterial enrichment and isolation as a part of the study about bacterial resource diversity in our lab [11]. A water sample, collected from Sayram Lake, Xinjiang Province, China (44° 30′ 30.41″ N, 81° 12′ 39.55″ E), was diluted stepwise using sterile distilled water and each diluted sample was spread evenly on marine agar 2216 (MA; Becton Dickinson, Franklin Lakes, NJ, USA). The strain XJSP^T^ was isolated from the coated medium, which was incubated at 25 °C for 10 days. Pure cultures were preserved for long-term in sterile 15% (*v*/*v*) glycerol supplemented with 1% (*w*/*v*) NaCl at –80 °C. The type of strain *P. sediminis* KCTC 72015^T^ was purchased as an experiment control strain from Korean Collection for Type Cultures center (KCTC). 

### 2.2. S rRNA Gene Sequencing and Phylogenetic Analysis 

The 16S rRNA genes of strain XJSP^T^ were amplified using polymerase chain reaction (PCR) technology with two universal primers for bacteria (27F and 1492R) and a purified gene product was cloned using the method described previously to obtain almost complete 16S rRNA gene sequence [12]. The 16S rRNA gene similarities between strain XJSP^T^ and closely related species were calculated using the NCBI BLAST service and EzBioCloud database. The 16S rRNA gene sequence of strain XJSP^T^ and those of relevant strains were aligned by MUSCLE service [13] and phylogenetic trees were reconstructed with 1000 bootstrap replicates based on neighbor-joining (NJ), minimum-evolution (ME), and maximum-likelihood (ML) algorithms in MEGA X software [14,15]. The integrated method T92 + G + I was calculated as the best-fit substitution pattern for reconstructing the ML tree. 

### 2.3. Whole-Genome Sequencing and Genome Annotation 

Purified genomic DNA was obtained employing the *SteadyPure* bacterial genomic DNA extraction kit (Accurate Biotechnology Co., Ltd., Hunan Province, China) following the user guide. The draft genome of strain XJSP^T^ was sequenced by Novogen (Tianjin, China) using Illumina Hiseq platform with the sequencing protocol of paired-end 150 bp fragment libraries and genome assembly was carried out with the Velvet software (v. 1.2.10) [16]. The genome sequences of related strains used in this paper were downloaded from the NCBI genomes repository. Gene prediction and annotation were carried out by Prodigal server [17] and the prokaryotic genome annotation pipeline (PGAP) implemented in NCBI [18]. 

### 2.4. Phylogenomic and Comparative Genomic Analysis 

Genome similarity indexes, average nucleotide identity (ANI), and DNA–DNA hybridization (DDH) were calculated employing the JSpeciesWS online service offered by Ribocon (https://jspecies.ribohost.com/jspeciesws/ (accessed on 13 July 2022)) [19] and genomes comparison calculator v. 3.0 (http://ggdc.dsmz.de/ggdc.php) [20], respectively. The IQ-TREE based on concatenated alignment sequences of 120 ubiquitous single-copy proteins was reconstructed by GTDB-Tk v. 1.3.0 with the LG+F+I+G4 pattern and 1000 bootstrap replicates [21,22,23]. To further investigate gene and protein differences among members of the genus *Pseudotabrizicola*, comparative genomic analysis was achieved by the ultra-fast bacterial pan-genome analysis tool (BPGA) with default parameters [24]. The analysis of metabolic pathways and the search of putative secondary metabolite biosynthetic gene clusters were accomplished by BlastKOALA service (v. 2.2) in Kyoto Encyclopedia of Genes and Genomes (KEGG, https://www.kegg.jp/blastkoala/ (accessed on 26 October 2022)) [25] and antiSMASH 6.0 (https://antismash.secondarymetabolites.org/ (accessed on 31 August 2022)) [26]. Photosynthetic genes *pufML* encoding for M and L subunit of core photosynthetic reaction center were detected in the genome based on the primer *pufL*-67F (5′-TTC GAC TTY TGG RTN GGNCC-3′) and *pufM*-781R (5′-CCA KSG TCC AGC GCC AGAANA-3′) using the software SnapGene v. 4.1.9 [27]. 

### 2.5. Phenotypic Characteristics 

Strain XJSP^T^ was incubated on MA medium at 33 °C for the implementation of phenotypic characteristics investigations. After culturing for four days, the Gram staining reaction was checked with the Gram-stain kit produced by bioMérieux company, and cell morphology was observed by employing light microscopy (E600, Nikon, Tokyo, Japan) and scanning electron microscopy (model Nova NanoSEM450, FEI). The growth of strains at various pH ranges (pH 5.5–9.5, at intervals of 0.5) was tested in marine broth 2216 (MB; Becton Dickinson, Franklin Lakes, NJ, USA) with various pH values, and growth status was quantified using a microplate reader at 600 nm. The pH of mediums was adjusted using commercial additional buffers at a concentration of 20 mM: MES (pH 5.5 and 6.0), PIPES (pH 6.5 and 7.0), HEPES (pH 7.5 and 8.0), Tricine (pH 8.5), and CAPSO (pH 9.0 and 9.5). Temperature conditions for growth were tested at 0, 4, 10, 15, 20, 25, 28, 30, 33, 37, 40, 42, and 45 °C for approximately 7 days on MA medium (growth was recorded every 12 h). Salt tolerance was assayed using modified MA (prepared according to the MA formula, but without NaCl) with different NaCl concentrations (0, 0.5, 1, 2, 3, 4, 5, 6, 7, 8, 9, and 10%, *w*/*v*). 

Oxidase test was examined by employing the commercial bioMérieux oxidase test kit, and catalase activity was detected through bubbles production after adding 3% (*v*/*v*) H_2_O_2_ to plate with fresh cultures. Hydrolysis tests of starch (0.2%, *w*/*v*), CM-cellulose (0.5%, *w*/*v*), alginate (2%, *w*/*v*), and Tweens (20, 40, 60, and 80, 1%, *v*/*v*) were determined based on the previous methods [28]. The commercial bioMérieux API 50CH and API ZYM reagent strips were used to test acid production and enzyme activities, respectively. Other biochemical analyses were performed applying the BIOLOG GEN III MicroPlates and API 50CH and all reagent strip tests were implemented following the user guide, except for adjusting the NaCl concentration to the optimum. The antimicrobial susceptibility test was investigated using the disc diffusion method under optimum conditions for a week [29]. 

Photoheterotrophic growth was tested under light exposure (2400 lx) and anaerobic conditions in the following liquid medium (per liter: 3 g sodium pyruvate, 1.2 g NH_4_Cl or 1g KNO_3_), prepared with modified artificial seawater (per liter of distilled water: 3.3 g MgSO_4_, 2.3 g MgCl_2_, 1.2 g CaCl_2_, 0.7 g KCl, 10 g NaCl) at 33 °C for 14 days. Photoautotrophic growth was determined by anaerobically incubating strain XJSP^T^ under light condition (2400 lx) with the following liquid medium (0.5 mM Na_2_S, 0.5 mM Na_2_S_2_O_3_ and 0.1% (*w*/*v*) NaHCO_3_), prepared with modified artificial seawater as described above [9]. Anaerobic conditions were achieved by boiling the liquid medium and adding sterilized liquid paraffin. Additionally, the presence of pigments was detected by in vitro spectrometric methods described by Biebl et al. [30]. Cells cultivated aerobically in MB medium for four days were collected, washed twice, and suspended in a mixture of acetone and methanol (7:2, *v*/*v*) to extract the pigments. Absorption spectra were measured using a spectrophotometer.

### 2.6. Chemotaxonomic Properties

Comparative analyses of chemotaxonomic property between Strain XJSP^T^ and experiment control strain *P. sediminis* KCTC 72015^T^ were performed using cells harvested in MB medium at the late stage of exponential growth phase. Lipids were obtained in the mixture system of chloroform, methanol, and water (2.5:5:2, *v*/*v*/*v*), and separated and identified by two-dimensional silica gel thin layer chromatography (TLC) plate [31,32]. Extracted fatty acids were separated and analyzed based on the TSBA40 database of the Sherlock Microbial Identification System (MIDI) by an Agilent gas chromatograph (product model 6890N), as used previously [33]. Respiratory quinones obtained from lyophilized thallus were separated by TLC plates and identified applying HPLC technology [34]. 

## 3. Results and Discussion

### 3.1. S rRNA Gene Sequence and Phylogenetic Analysis

**Figure 1:**
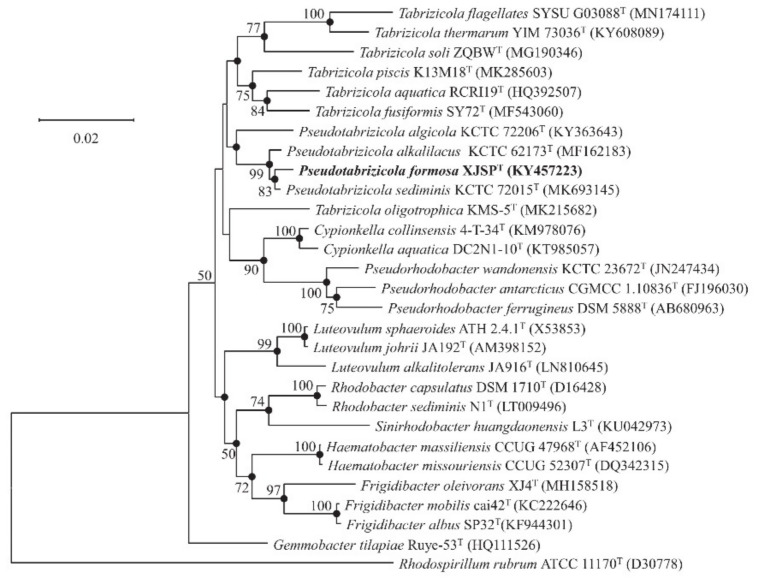
Neighbor-joining phylogenetic tree based on 16S rRNA gene sequences of strain XJSP^T^ and other closely related species. Filled circles indicate branches that were recovered with all three methods (neighbor-joining, maximum-likelihood, and minimum-evolution). Percentages bootstrap values above 50% (1000 replicates) are shown at branch nodes. *Rhodospirillum rubrum* ATCC 11170^T^ was used as the out-group. Bar, 0.02 substitutions per nucleotide position.

Almost complete 16S rRNA gene sequence of strain XJSP^T^ (1425 bp) was obtained in this study. The 16S rRNA gene sequence similarity values between Strain XJSP^T^ and members of the genus *Pseudotabrizicola* showed 97.7–99.5% (Table 1). The NJ tree inferred from 16S rRNA gene sequence exhibited strain XJSP^T^ located in the cluster of *Pseudotabrizicola* species, which supported strain XJSP^T^ belonged to the genus *Pseudotabrizicola* (Figure 1). The topology of strain XJSP^T^ and the genus *Pseudotabrizicola* was also obtained in the phylogenetic trees reconstructed with the ML and ME algorithm (Figure 1). 

### 3.2. Genome Properties and Phylogenetic Analysis

The draft genome (strain XJSP^T^) of 3,702,758 bp in length was obtained after assembly with an average 300× coverage depth, producing 14 contigs, and the N50 value is 812,613 bp. All contigs were larger than 1595 bp, with the largest being 1,520,968 bp. The calculated G+C content was estimated to be 63.4 mol%. The 16S rRNA gene sequence of strain XJSP^T^ detected from genome (1467 bp) covered that obtained by amplification (1425 bp). The PGAP results showed that a total of 3552 genes were predicted, including 52 RNA genes (3 rRNA genes, 3 ncRNA genes, and 46 tRNA genes) and 3470 potential protein-coding genes. Detailed comparison results of genome statistics of the *Pseudotabrizicola* are shown in Table 2. 

**Figure 2:**
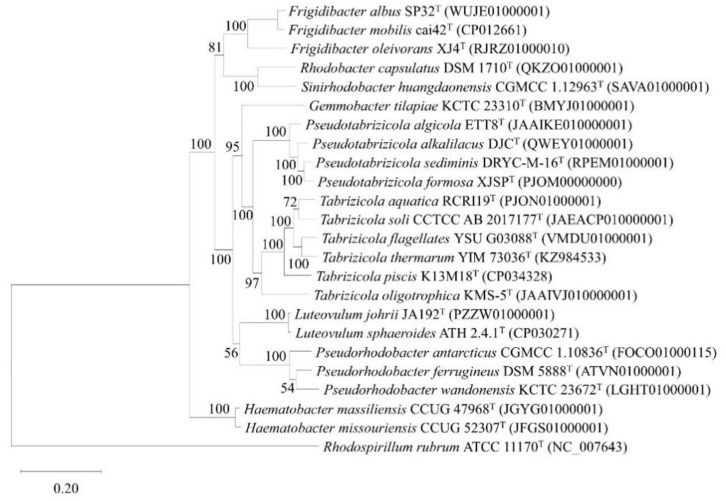
The IQ-TREE based on 120 ubiquitous single-copy proteins. Percentages bootstrap values (1000 replicates) are shown at branch nodes. *Rhodospirillum rubrum* ATCC 11170^T^ was used as the out-group. Bar, 0.20 substitutions per nucleotide position.

The genome similarity indices ANI and DDH values between strain XJSP^T^ and *Pseudotabrizicola* species were 81.4–87.3% and 23.9–32.5% respectively, with both being lower than the values for species demarcation [35,36] (Table 1), which indicated that strain XJSP^T^ was a novel member belonging to the genus *Pseudotabrizicola*. The IQ-TREE built on concatenated alignment sequences of 120 ubiquitous single-copy proteins in bacteria showed the evolutionary relationships of strain XJSP^T^ and the genus *Pseudotabrizicola* (Figure 2). 

### 3.3. Pan-Genome Analysis of the Genus Pseudotabrizicola 

**Figure 3:**
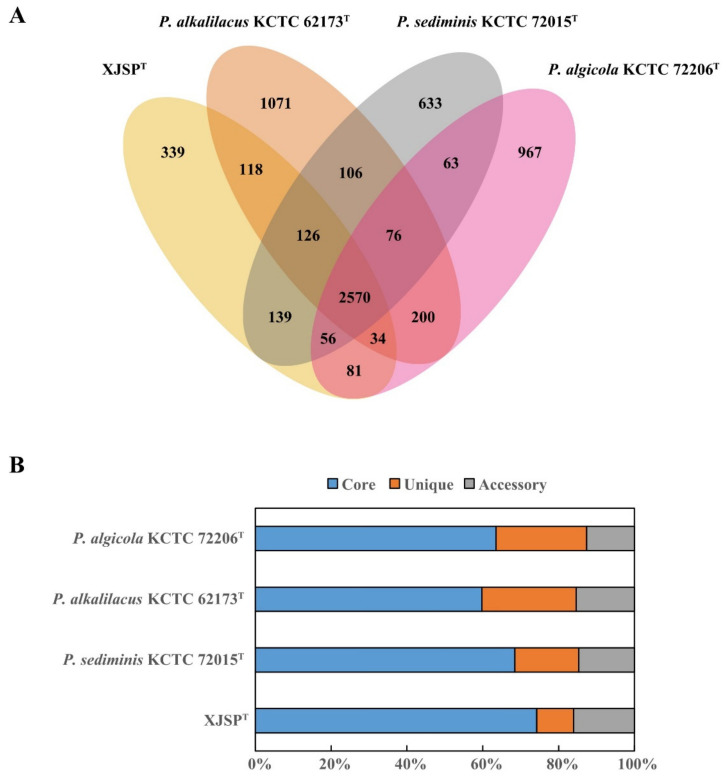
Comparisons of *Pseudotabrizicola* orthologous protein groups in four *Pseudotabrizicola* genomes. (**A**) Venn diagram displaying the numbers of core gene families and unique genes for each of the four *Pseudotabrizicola* strains. (**B**) Percentage of core, accessory, and unique genes in each of the four genomes.

Comparative genomic analysis of the genus *Pseudotabrizicola* was carried out to identify the consistency and difference of the members. As shown in Figure 3, 2570 core genes were shared by the four *Pseudotabrizicola* species, strain XJSP^T^, *P. sediminis* KCTC 72015^T^, *P. alkalilacus* KCTC 62173^T^, and *P. algicola* KCTC 72206^T^, which accounted for more than half (59.8–74.2%) of each genome. KEGG annotation was performed for core, accessory, and unique genes to analyze their distribution in different metabolic pathways. The results showed that the core genes were more involved in the metabolisms of amino acid, energy and nucleotide, translation and replication, and repair. The proportion of accessory genes was higher than that of core genes and unique genes in carbohydrate metabolism, cofactors and vitamins metabolism and xenobiotics biodegradation. However, unique genes contributed more to drug resistance, lipid metabolism, membrane transport, and signal transduction (Supplementary Figure S1). 

### 3.4. Metabolic Pathways and Secondary Metabolites Analyses 

**Figure 4:**
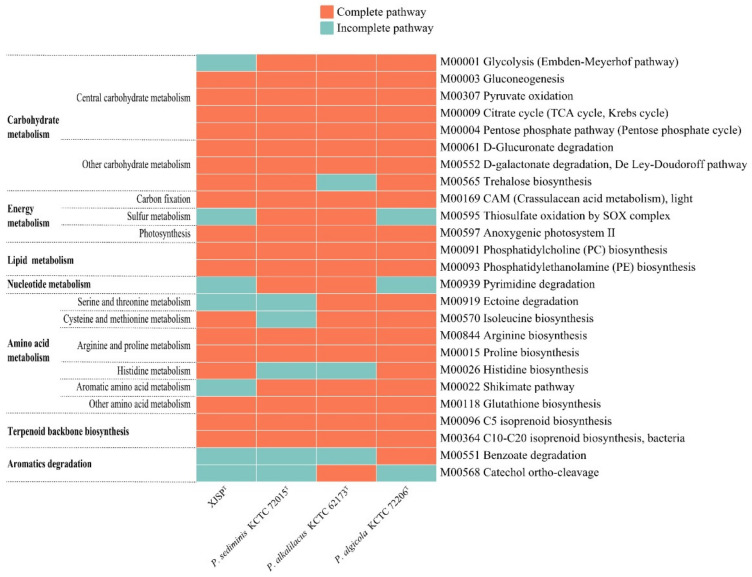
Heat maps of complete and incomplete metabolic pathways in the genomes of strain XJSP^T^, *P. sediminis* KCTC 72015^T^, *P. alkalilacus* KCTC 62173^T^, and *P. algicola* KCTC 72206^T^.

The results of metabolic pathways analyzed by KEGG’s BlastKOALA service showed that most of carbohydrate metabolism pathways were intact, except for the incomplete glycolysis pathway (M00001) in strain XJSP^T^. The four species of the genus *Pseudotabrizicola*, strain XJSP^T^, *P. sediminis* KCTC 72015^T^, *P. alkalilacus* KCTC 62173^T^, and *P. algicola* KCTC 72206^T^, all possessed a complete anoxygenic photosystem II (M00597), namely the L and M subunits of photosynthetic reaction center. The *pufML* gene sequences of strain XJSP^T^ are given in Supplementary Table S1. Moreover, strain XJSP^T^ had complete phosphatidylcholine (PC) and phosphatidylethanolamine (PE) biosynthesis pathway (M00091 and M00093, respectively) and isoprenoid biosynthesis pathway (M00096 and M00364), which was consistent with *P. sediminis* KCTC 72015^T^, *P. alkalilacus* KCTC 62173^T^, and *P. algicola* KCTC 72206^T^ (Figure 4). The potential secondary metabolites synthesized by strain XJSP^T^ were identified using antiSMASH. The results showed that the genome of strain XJSP^T^ encoded eight identified gene clusters about the biosynthesis of secondary metabolites (Supplementary Table S2). One of the eight gene clusters, for terpene, showed 100% similarity to a known biosynthetic gene cluster-encoding carotenoid [37]. 

### 3.5. Phenotypic Characteristics

Colonies of strain XJSP^T^ were cream-colored, smooth, and circular after incubating for 3 days at 33 °C on MA medium, and the color of colonies would change to light opaque-pink after a week under a low light condition (10 µmol photons m^−2^ s^−2^). Cells of strain XJSP^T^ were Gram-stain-negative, and rod-shaped with widths of 0.3–0.5 μm and lengths of 0.8–2.0 μm (Supplementary Figure S2). Strain XJSP^T^ was unable to undergo autotrophic and heterotrophic growth under light and anaerobic conditions. The activities of esterase (C4), esterase lipase (C8) and leucine arylamidase were positive but ɑ-galactosidase activity was negative, which was consistent with *P. sediminis* KCTC 72015^T^ and *P. alkalilacus* KCTC 62173^T^ [38]. However, there were several characteristic differences between strain XJSP^T^ and related species summarized in Table 3, which could distinguish strain XJSP^T^ from related species. Spectral analysis showed that the typical maxima absorptions were at 486, 867, and 895 nm, which indicated the presence of BChl a and carotenoids (Supplementary Figure S3). Strain XJSP^T^ was found to be sensitive to (μg per disc) chloramphenicol (30), rifampicin (5), cefotaxime sodium (30), ceftriaxone (30), acetylspiramycin (30), clarithromycin (15), tobramycin (10), ampicillin (10), norfloxacin (30), and neomycin (30), but resistant to penicillin (10), erythromycin (15), tetracycline (30), vancomycin (30), lincomycin (2), gentamycin (10), nalidixic acid (30), streptomycin (10), and kanamycin (30). 

### 3.6. Chemotaxonomic Properties 

The isoprenoid quinone detected in strain XJSP^T^ was Q-10, which was in line with the genus *Pseudotabrizicola*. The major cellular fatty acids (>10%) were iso-C_18:0_ and summed feature 8 (comprising C_18:1_ ω6c and/or C_18:1_ ω7c) (Supplementary Table S3). The major polar lipids of strain XJSP^T^ were phosphatidylcholine (PC), phophatydilethanolamine (PE), phosphatidylglycerol (PG), one unidentified phospholipid (PL), and one unknown lipid (L). The polar lipids composition of strain XJSP^T^ was similar to that of *P. sediminis* KCTC 72015^T^ in phosphatidylcholine (PC), phosphatidylglycerol (PG), and phophatydilethanolamine (PE), but the absence of diphosphatidylglycerol (DPG) in strain XJSP^T^ distinguished it from the closest strain *P. sediminis* KCTC 72015^T^ (Supplementary Figure S4). 

Description of *Pseudotabrizicola formosa* sp. nov.

*Pseudotabrizicola formosa* (for.mo’sa. L. fem. adj. *formosa* beautiful, beautifully formed, finely formed).

Cells are Gram-stain-negative and rod-shaped (0.3–0.5 µm wide and 0.8–2.0 µm long). Colonies appear cream-colored or light pink, circular with entire edges, and convex with a diameter of 1.0–1.5 mm. The cell suspension is a light opaque-pink in color. Cells growth occurs at 4–40 °C (optimum 33 °C), with 0–6.0% (*w*/*v*) NaCl (optimum 1.0% NaCl) and pH 6.5–9.5 (optimum pH 7.5). Phototrophic growth occurs under aerobic, heterotrophic conditions, and photosynthetic pigments are produced in low light (10 µmol photons m^−2^ s^−2^). The activities of catalase, oxidase, and valine arylamidase are positive but the activities of lipase (C14), N-acetyl-β-glucosaminidase, trypsin, α-mannosidase, α-chymotrypsin, acid phosphatase, and β-glucuronidase are negative. Hydrolyses of Tweens 20 and 40 are positive, but negative for hydrolyses of Tween 60, Tween 80, starch, CM-cellulose, alginate, and casein. Acids are produced from d-ribose, d-cellobiose, d-xylose, d-turanose, and d-galactose. In the oxidation test of sole carbon source, positive for d-maltose, d-trehalose, d-cellobiose, gentiobiose, sucrose, d-turanose, β-methyl-d-glucoside, d-salicin, d-mannose, d-fructose, l-fucose, myo-inositol, and l-malic acid. The major cellular fatty acids (>10%) are iso-C_18:0_ and summed feature 8 (comprising C_18:1_ ω6c and/or C_18:1_ ω7c). The main respiratory quinone is Q-10. The predominant polar lipids consist of phosphatidylcholine, phosphatidylglycerol, phophatydilethanolamine, one unidentified phospholipid, and one unknown lipid.

The type strain, XJSP^T^ (= KCTC 52636^T^ = MCCC 1H00184^T^ = SDUM 107003^T^), was isolated from Sayram Lake water, Xinjiang Province, China. The DNA G+C content of type strain was 63.4 mol%.

## Figures and Tables

**Table 1:** Comparisons of the 16S rRNA gene sequence similarity, average nucleotide identity (ANI), and DNA–DNA hybridization (DDH) values between strain XJSP^T^ and members of the genus *Pseudotabrizicola*.

Strains	XJSP^T^		
	16S rRNA Gene Similarity (%)	ANI (%)	DDH (%)
*P. sediminis* KCTC 72015^T^	99.5	87.3	32.5
*P. alkalilacus* KCTC 62173^T^	99.1	83.4	26.3
*P. algicola* KCTC 72206^T^	97.7	81.4	23.9

**Table 2:** Genome statistics of strain XJSP^T^ and members of the genus *Pseudotabrizicola*.

	1	2	3	4
Genome size (bp)	3,702,758	4,040,697	4,610,061	4,491,281
Contigs	14	86	110	29
N50 length (bp)	812,613	296,200	284,500	694,770
G+C content (mol %)	63.4	63.0	62.9	64.4
Genes	3552	3897	4476	4391
Protein-coding genes	3470	3755	4337	4253
tRNA genes	46	43	49	46
rRNA genes	3	3	7	3
ncRNA genes	3	3	3	3
GenBank ID	PJOM 00000000	NZ_RPEM 00000000	NZ_QWEY 00000000	NZ_JAAIKE 000000000

Strains: 1, XJSP^T^; 2, *P. sediminis* KCTC 72015^T^; 3, *P. alkalilacus* KCTC 62173^T^; 4, *P. algicola* KCTC 72206^T^.

**Table 3:** Differential characteristics between strain XJSP^T^ and related species.

Characteristic	1	2	3 ^a^
**Colony color**	cream or light pink	opaque-pink	cream
**Temperature range (°C** **)**	4–40	4–35	15–37
**NaCl range (%, *w*/*v*)**	0–6.0	1.0–2.0	0–3.0
**pH range**	6.5–9.5	7.0–9.0	6.0–10.0
**Voges–Proskauer reaction**	+	−	+
**Enzyme activity:**			
Arginine dihydrolase	−	−	+
Urease	−	−	+
Alkaline phosphatase	−	+	+
Cystine arylamidase	−	−	+
ɑ-glucosidase	+	−	+
β-glucosidase	−	−	+
β-galactosidase	−	−	+
**Hydrolysis of:**			
Tween 20	+	−	NA
Tween 40	+	−	+
**Acid production from:**			
Glycerol	−	+	+
d-tagatose	−	−	w
d-arabitol	−	+	NA
**Oxidation of:**			
ɑ-d-glucose	+	w	+
Inosine	+	+	−
d-glucose-6-PO4	+	−	+
**Major fatty acids (>10%)**	iso-C18:0, Summed feature 8	iso-C18:0, Summed feature 8	Summed feature 8
**Polar lipids**	PC, PG, PE, PL, L	PC, PG, DPG, PE, APL, AL, PL, L	PG, DPG, PE, PL, L
**DNA G+C content (mol %)**	63.4	63.0	62.9

Strains: 1, XJSP^T^; 2, *P. sediminis* KCTC 72015^T^; 3, *P. alkalilacus* KCTC 62173^T^. All data were from this study unless indicated otherwise. +, positive; −, negative; w, weakly positive; NA, no data available. Summed features are groups of two or three fatty acids that cannot be separated by GLC using the MIDI system. Summed feature 8 comprised C_18:1_ ω7c and/or C_18:1_ ω6c. PC, phosphatidylcholine; PG, phosphatidylglycerol; DPG, diphosphatidylglycerol; PE, phosphatidylethanolamine; APL, unidentified aminophospholipid; AL, unidentified aminolipid; PL, unidentified phospholipid; L, unidentified lipid. ^a^ Data from Phurbu et al. (2019) [38].

## Data Availability

The 16S rRNA gene sequence of *Pseudotabrizicola formosa* XJSP^T^ has been deposited at GenBank database with the accession number KY457223. The GenBank accession number for draft genome sequence of *Pseudotabrizicola formosa* XJSP^T^ is PJOM00000000.

## Supplementary Materials

The following supporting information can be downloaded at: https://www.mdpi.com/article/10.3390/microorganisms10112154/s1, Figure S1: The distribution of core genes, accessory genes and unique genes to different metabolic pathways in the genus *Pseudotabrizicola*. Figure S2: Scanning electron micrograph of cells of strain XJSP^T^. Bar, 5 μm. Figure S3: Absorption spectrum of the acetone/methanol (7:2) extract of strain XJSP^T^. The typical maxima absorptions at 486, 867 and 895 nm, indicating the presence of bacteriochlorophyll α and carotenoids. Figure S4: Two-dimensional TLC plate image of the total polar lipids of strain XJSP^T^ (a) and *Pseudotabrizicola sediminis* KCTC 72015^T^ (b). PC, phosphatidylcholine; PG, phosphatidylglycerol; DPG, diphosphatidylglycerol; PE, phosphatidylethanolamine; APL, unidentified aminophospholipid; AL, unidentified aminolipid; PL, unidentified phospholipid; L, unidentified lipid. Table S1: *pufML* gene sequences of strain XJSP^T^. Table S2: Secondary metabolites of strain XJSP^T^ predicted by antiSMASH. Table S3: Cellular fatty acid composition (%) of strain XJSP^T^ and related species.

## Abbreviations

AAPB	Aerobic anoxygenic phototrophic bacteria
ANI	Average nucleotide identity
DDH	DNA–DNA hybridization
MCCC	Marine Culture Collection of China
SDUM	Shandong University Collection of Marine Microorganisms
KCTC	Korean Collection for Type Cultures
PGAP	Prokaryotic Genome Annotation Pipeline
BPGA	Bacterial Pan-Genome Analysis Tool
MES	2-Morpholinoethanesulfonic acid
PIPES	1,4-Piperazinediethanesulfonic acid
HEPES	N-(2-Hydroxyethyl) piperazine-N’-2-ethanesulfonic acid
CAPSO	3-(Cyclohexylamino)-2-hydroxy-1-propanesulfonic acid
Tricine	N-[Tris(hydroxymethyl)methyl]glycine
