# Supplementary material for: Genomic Analysis and Characterization of Pseudotabrizicola formosa sp. nov., a Novel Aerobic Anoxygenic Phototrophic Bacterium, Isolated from Sayram Lake Water
Source: Microorganisms. 2022 Oct 30;10(11):2154. doi: 10.3390/microorganisms10112154 (PMC9698765; doi:10.3390/microorganisms10112154)
Supplement: Supplementary file 1 [file microorganisms-10-02154-s001.zip › microorganisms-1994636-supplementary.pdf]

Supplementary Materials

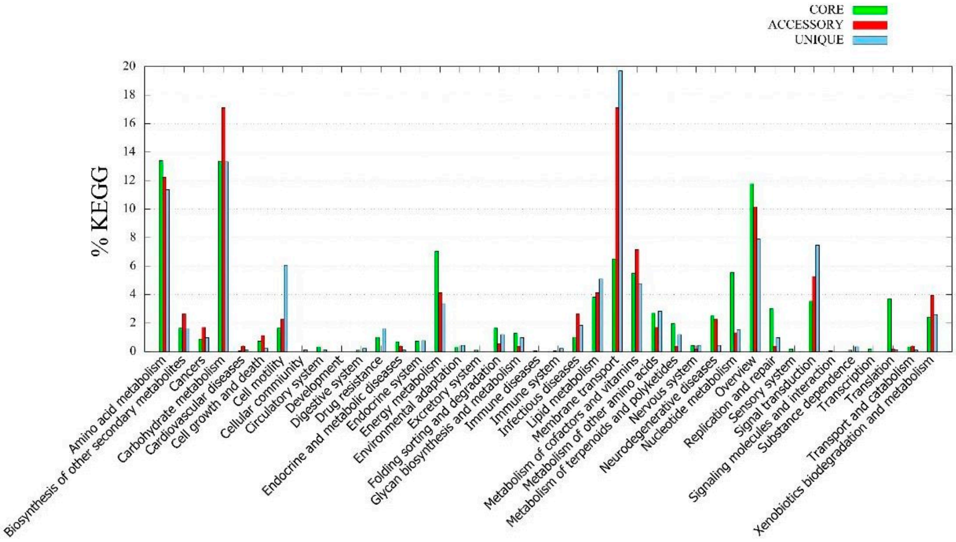

Figure S1. The distribution of core genes, accessory genes and unique genes to different metabolic pathways in the genus *Pseudotabrizicola.*

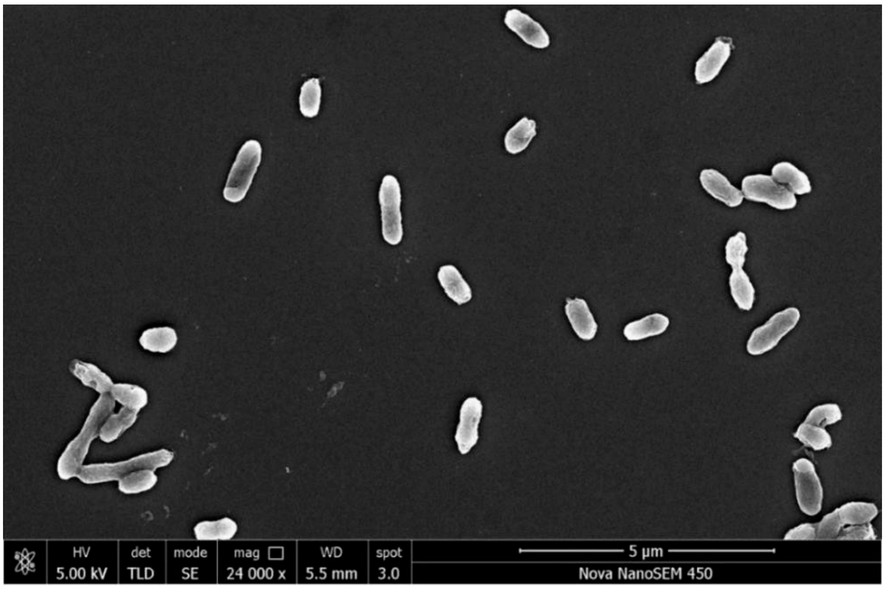

Figure S2. Scanning electron micrograph of cells of strain XJSP^{T}. Bar, 5 µm.

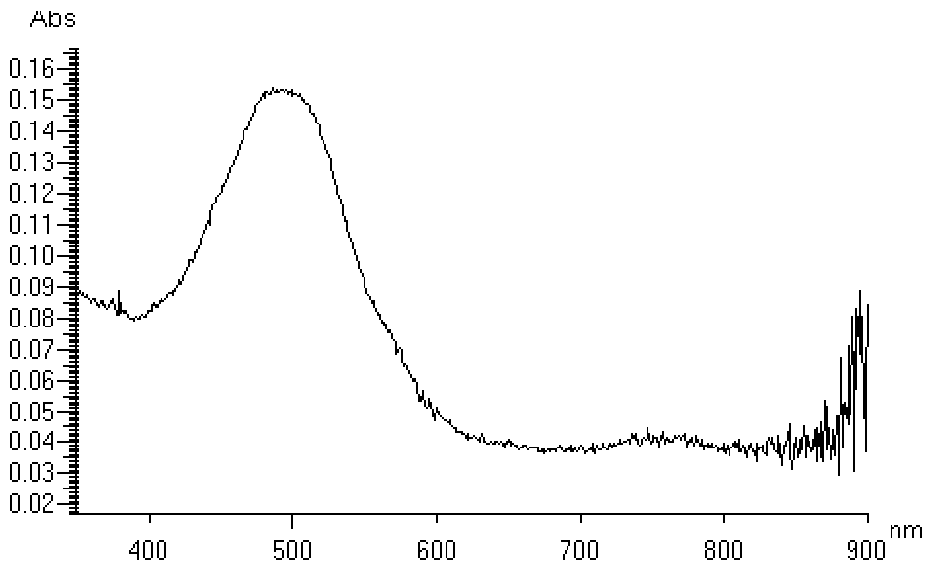

Figure S3. Absorption spectrum of the acetone/methanol (7:2) extract of strain XJSP^T.The typical maxima absorptions at 486, 867 and 895 nm, indicating the presence of bacteriochlorophyll α and carotenoids.

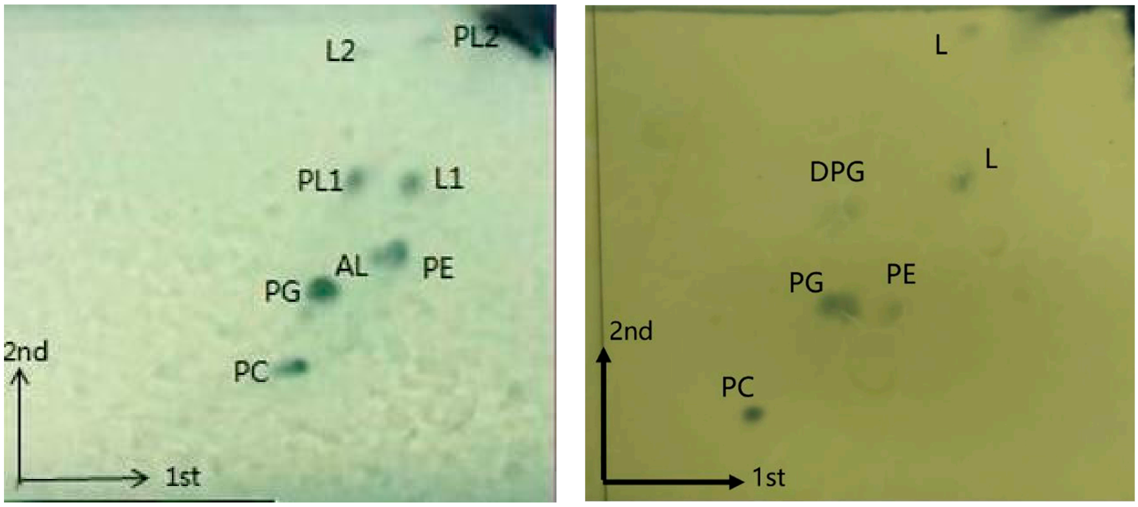

(**a**) (**b**)

Figure S4. Two-dimensional TLC plate image of the total polar lipids of strain XJSP^T (**a**) and *Pseudotabrizicola sediminis* KCTC 72015^T (**b**). PC, phosphatidylcholine; PG, phosphatidylglycerol; DPG, diphosphatidylglycerol; PE, phosphatidylethanolamine; APL, unidentified aminophospholipid; AL, unidentified aminolipid; PL, unidentified phospholipid; L, unidentified lipid.

Table S1. *pufML* gene sequences of strain XJSP^T.

Strain	*pufML* gene sequences
XJSP^T	TTCGACTTCTGGGTCGGCCCTTTCTACGTCGGCTTCTTTGGAGTCGTGGGCT<br>TCTTCTTTGCAGCCCTCGGCACGATTCTGATATTCTACGGCGCGAGCCTGCA<br>GGGGACATGGAACCCCTGGCTGATCTCGATCGCGCCGCCCCCTGTCGAAAT<br>GGGCCTGGCTGCCGCACCTTTGCGCGACGGCGGGCTGTGGCAGATCATCAC<br>CATCTGCGCGACCGGGGCCTTTACGGCCTGGGCGCTGCGCGAGGTGGAAAT<br>CTGCCGCAAGCTGGGCATGGGCTATCATGTGCCCGTGGCCTTCGGGGTGGCG<br>ATCTTTGCCTACCTCACGCTGGTGGTGATCCGCCCGGTCATGATGGGCGCCT<br>GGGGCTATGCCTTTCCCTACGGGATCTGGACGCACCTCGACTGGGTGTCGAA<br>CACCGGCTACCAATATGGCAACTTCCACTACAACCCGGCGCATATGATCGCG<br>GTGAGCTTTTTCTTCACCACGGCGCTGGCCTTGTCCTTGCACGGCGCGCTGA<br>TCCTGTCGGCGGCCAATCCCGAAAAGGGCAAGGAAATGCGGACCCCAGATC<br>ACGAGGATACCTATTTCCGCGATCTGATCGGGTATTCCGTCGGCACGCTGGG<br>CATCCACCGTGTGGGCCTGCTGCTGGCCCTGAACGCCGGGTTCTGGTCGGC<br>AATCTGTATCGTTATCTCCGGCACCATCTGGTTCGATCAATGGATCGTCTGGT<br>GGGACTGGTGGCTGAACCTGCCTTGGTGGGCCGGTATCGAGGGAGGCATCA<br>ATGGCTGAGTATCAAAACATCTTCACTCAGGTCCAGGTCCGCGCCGAGCCCG<br>AAATGGGTCTTGTCGAAGGGGTGGAACTGCACAACCGCACCAGCGGTGCC<br>GGGTTCTCGAACCTTGCCGGCTGGATCGGCAACGCCCAGCTGGGGCCGGTC<br>TATCTGGGCACCATGGGCGTGATCTCGCTGGTGTCGGGGGCGATCTGGTTCT<br>TCACCGTCGGCGCCTGGTACTGGTATCAGGCGGGTCTGAACCCGGCCGTGTT<br>CCTGCGCGACCTGTTCTGGTTCAGCCTTGAGCCACCGGCCGAGGAATATGGT<br>CTGGGCTTTGCCCCGATTGCCGAAGGGGGGCTGTGGATCATTGCCAGCTTCT<br>TCCTGCTGGTCTCTGTCTGCGCCTGGTGGGTGCGCACCTACCTTCGGGCGCA<br>AGCGCTGGGGATGGGCAAGCATGTGTCCTGGGCCTTTGCCTCGGCCATCTG<br>GTTGTTCCTGGTTCTGGGCCTGTTCCGTCCGATCCTGATGGGATCGTGGAGC<br>CACGCCGTCCCTTATGGCATCTTCAGCCATCTGGACTGGACCAACCTGTTCA<br>GCCTGACCTATGGCAACCTGTTCTATAACCCGTTCCACGCGCTTTCGATCGCG<br>TTCCTCTATGGCTCGGCCCTGCTGTTTGCCATGCATGGGGCGACCATCCTGGC<br>GGTGTCCCGTTTCGGCGGTGACCGCGAGCTGGAGCAGATCGTTGACCGTGG<br>CACGGCCTCCGAACGGGCGGCGCTGTTCTGGCGCTGGACCATGG

Table S2. Secondary metabolites of strain XJSP^T predicted by antiSMASH.

Region	Type	Gene Count
1	RiPP-like [a]	12
2	NRPS [b]	30
3	Terpene	17
4	Terpene	17
5	RRE-containing [c]	19
6	T1PKS [d]	44
7	Ectoine	18
8	Hserlactone [e]	21

[a] Other unspecified ribosomally synthesised and post-translationally modified peptide product (RiPP) cluster; [b] Non-ribosomal peptide synthetase cluster; [c] RRE-element containing cluster; [d] Type I PKS (Polyketide synthase) cluster; [e] Homoserine lactone cluster.

Table S3. Cellular fatty acid composition (%) of strain XJSP^T and related species.

**Fatty acid**	**1**	**2**
$C_{16:0}$	TR	3.0
$C_{18:0}$	–	2.6
cyclo-$C_{19:0}$ ω8c	–	1.6
iso-$C_{18:0}$	**12.2**	**12.8**
$C_{10:0}$ 3–OH	–	1.8
$C_{18:0}$ 3–OH	2.3	2.3
3[a]	TR	1.1
7[a]	2.0	TR
8[a]	**75.2**	**69.5**

Strains: 1, XJSP^T; 2, *P. sediminis* KCTC 72015^T. All data listed in the table are from this study. TR, trace (<1.0%); −, Not detected; Fatty acids present at >10% are indicatedin bold.

[a] Summed features are groups of two or three fatty acids that cannot be separated by GLC using the MIDI system. Summed feature 3 comprised $C_{16:1}$ ω6c and/or $C_{16:1}$ ω7c, Summed feature 7 comprised C1$_{9:1}$ ω7c/$C_{19:1}$ ω6c and/or $C_{19:1}$ ω6c/ω7c/19cy and summed feature 8 comprised $C_{18:1}$ ω6c and/or $C_{18:1}$ ω7c
